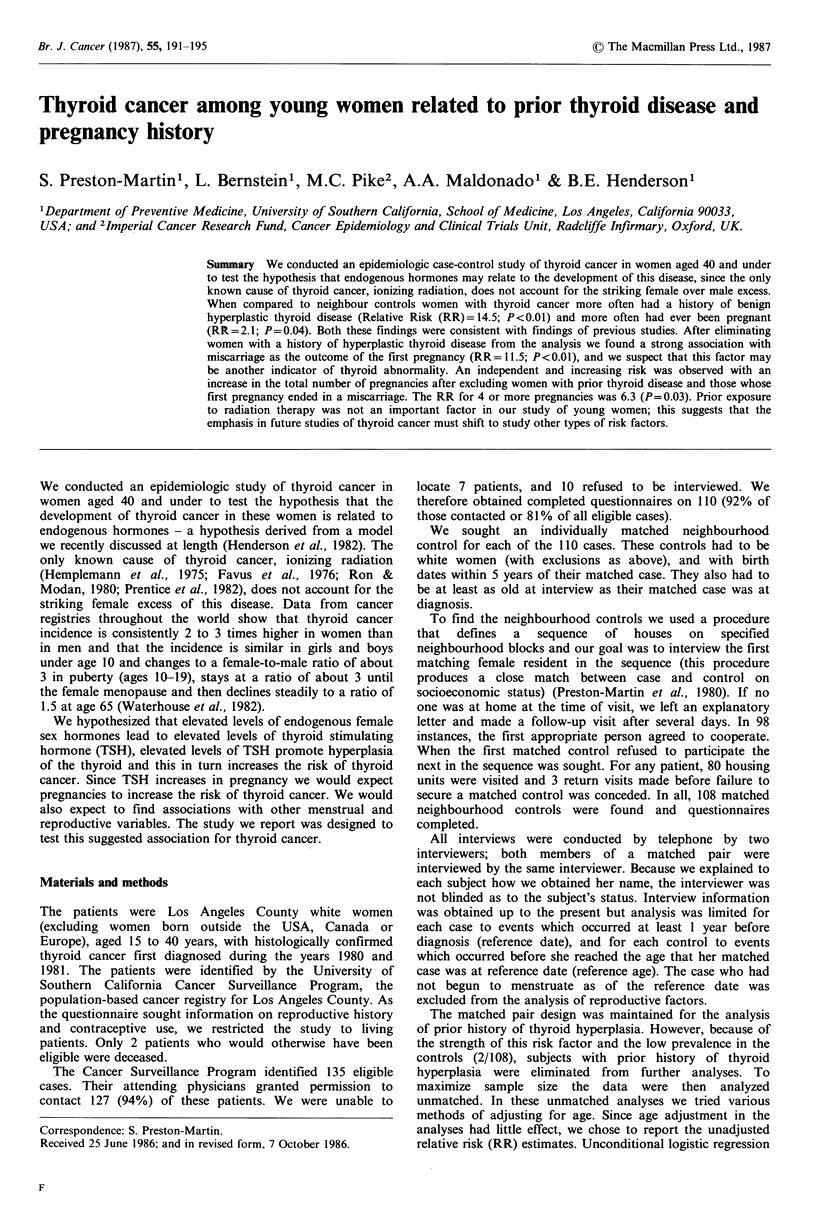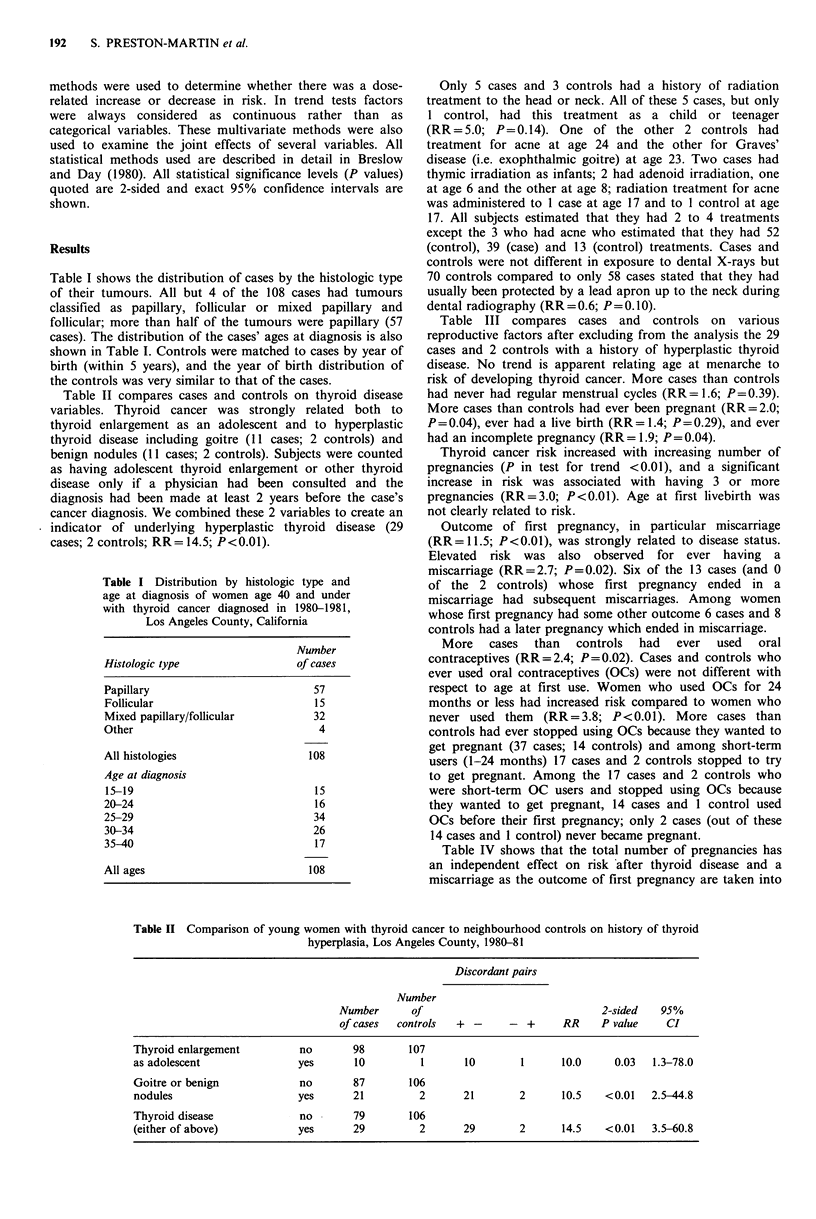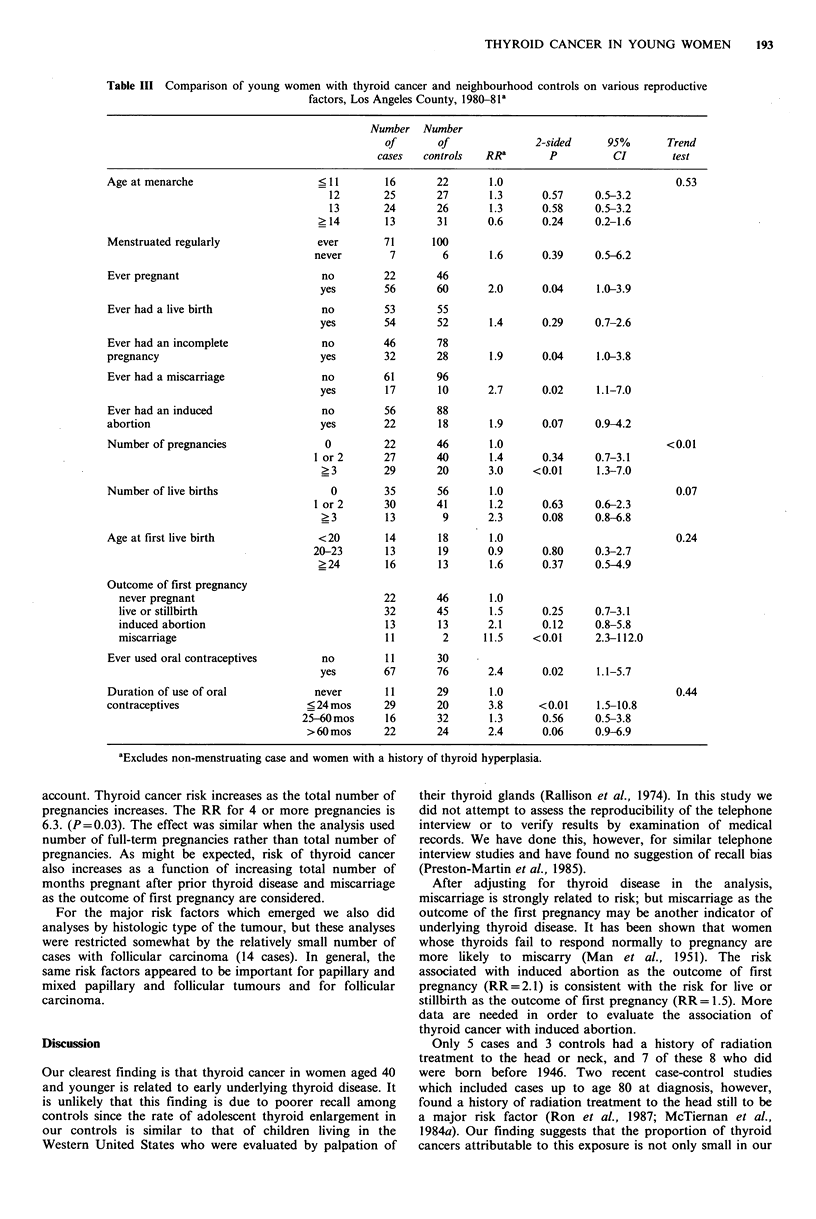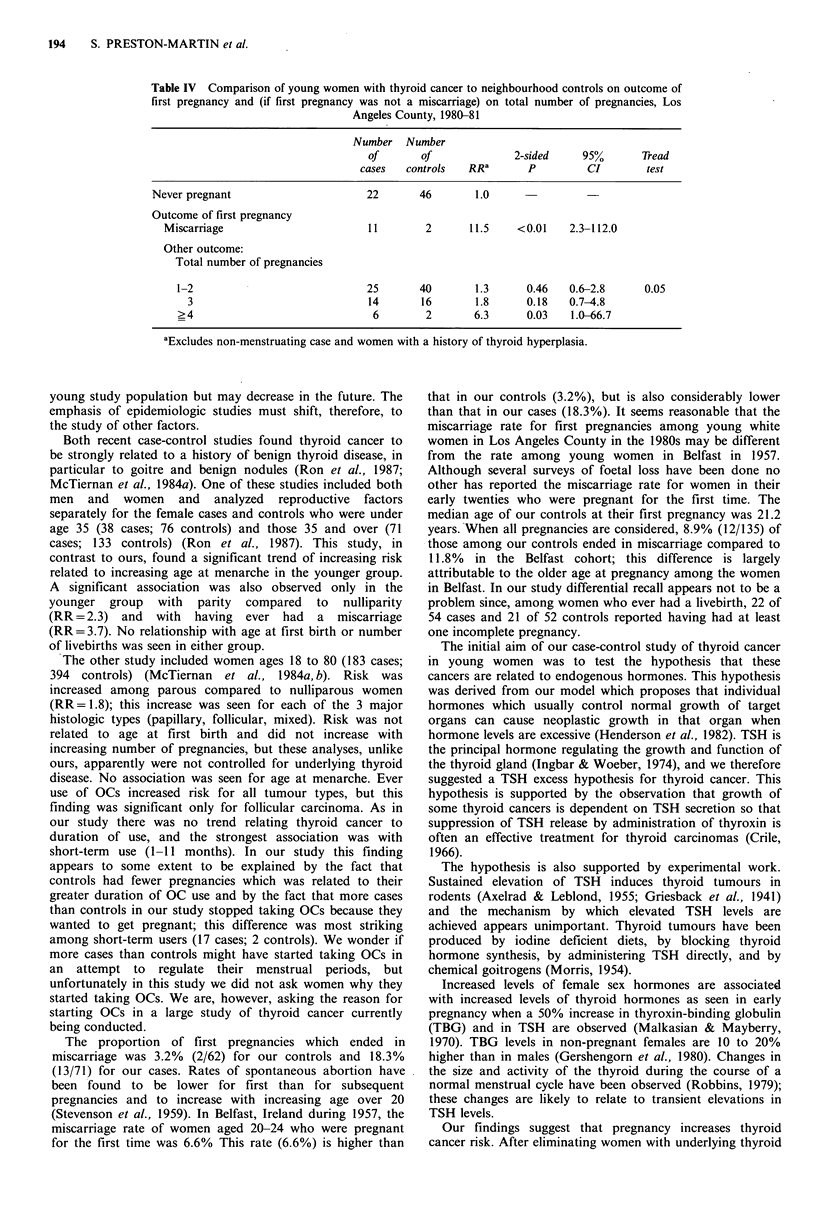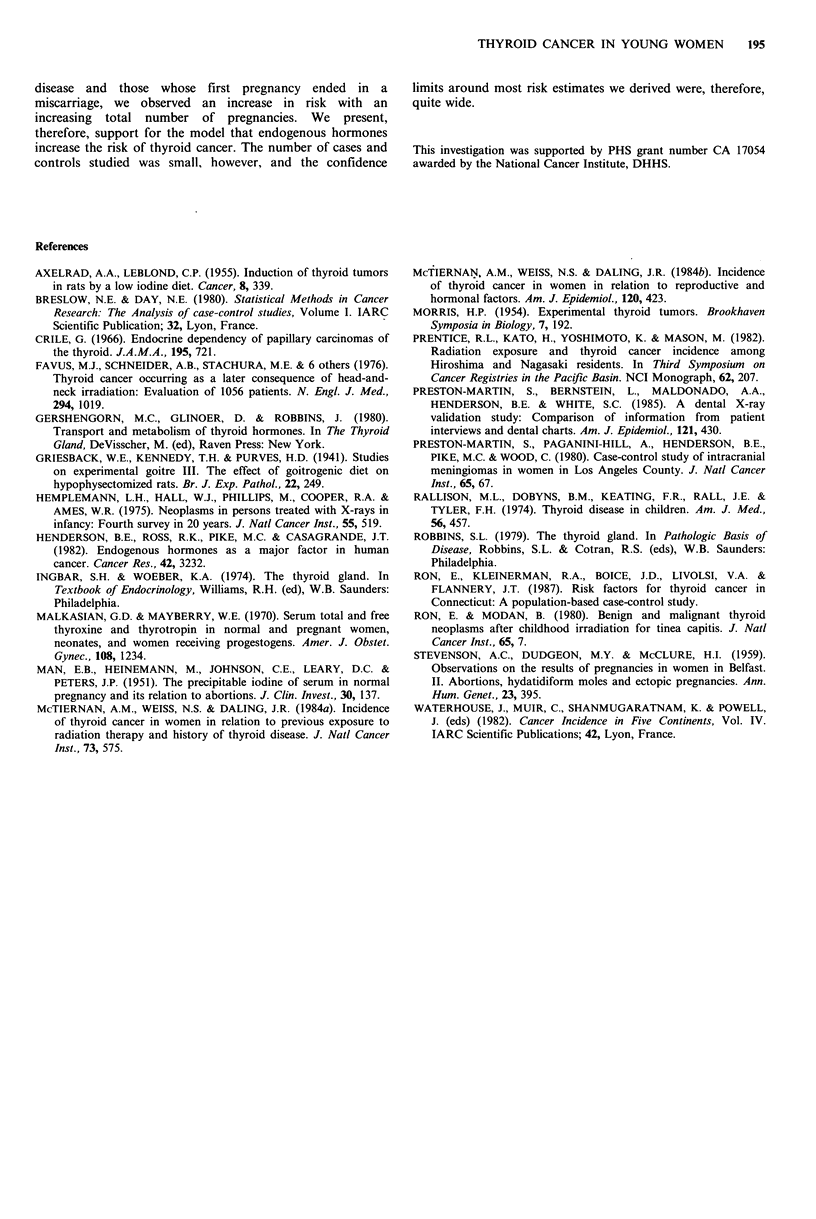# Thyroid cancer among young women related to prior thyroid disease and pregnancy history.

**DOI:** 10.1038/bjc.1987.36

**Published:** 1987-02

**Authors:** S. Preston-Martin, L. Bernstein, M. C. Pike, A. A. Maldonado, B. E. Henderson

## Abstract

We conducted an epidemiologic case-control study of thyroid cancer in women aged 40 and under to test the hypothesis that endogenous hormones may relate to the development of this disease, since the only known cause of thyroid cancer, ionizing radiation, does not account for the striking female over male excess. When compared to neighbour controls women with thyroid cancer more often had a history of benign hyperplastic thyroid disease (Relative Risk (RR) = 14.5; P less than 0.01) and more often had ever been pregnant (RR = 2.1; P = 0.04). Both these findings were consistent with findings of previous studies. After eliminating women with a history of hyperplastic thyroid disease from the analysis we found a strong association with miscarriage as the outcome of the first pregnancy (RR = 11.5; P less than 0.01), and we suspect that this factor may be another indicator of thyroid abnormality. An independent and increasing risk was observed with an increase in the total number of pregnancies after excluding women with prior thyroid disease and those whose first pregnancy ended in a miscarriage. The RR for 4 or more pregnancies was 6.3 (P = 0.03). Prior exposure to radiation therapy was not an important factor in our study of young women; this suggests that the emphasis in future studies of thyroid cancer must shift to study other types of risk factors.


*Br. J. Cancer* (1987), **55**, 191–195 © The Macmillan Press Ltd., 1987

### Thyroid cancer among young women related to prior thyroid disease and pregnancy history

S. Preston-Martin[1], L. Bernstein[1], M.C. Pike[2], A.A. Maldonado[1] & B.E. Henderson[1]

[1]*Department of Preventive Medicine, University of Southern California, School of Medicine, Los Angeles, California 90033, USA; and* [2]*Imperial Cancer Research Fund, Cancer Epidemiology and Clinical Trials Unit, Radcliffe Infirmary, Oxford, UK.*

**Summary** We conducted an epidemiologic case-control study of thyroid cancer in women aged 40 and under to test the hypothesis that endogenous hormones may relate to the development of this disease, since the only known cause of thyroid cancer, ionizing radiation, does not account for the striking female over male excess. When compared to neighbour controls women with thyroid cancer more often had a history of benign hyperplastic thyroid disease (Relative Risk (RR) = 14.5; $P<0.01$) and more often had ever been pregnant (RR = 2.1; $P=0.04$). Both these findings were consistent with findings of previous studies. After eliminating women with a history of hyperplastic thyroid disease from the analysis we found a strong association with miscarriage as the outcome of the first pregnancy (RR = 11.5; $P<0.01$), and we suspect that this factor may be another indicator of thyroid abnormality. An independent and increasing risk was observed with an increase in the total number of pregnancies after excluding women with prior thyroid disease and those whose first pregnancy ended in a miscarriage. The RR for 4 or more pregnancies was 6.3 ($P=0.03$). Prior exposure to radiation therapy was not an important factor in our study of young women; this suggests that the emphasis in future studies of thyroid cancer must shift to study other types of risk factors.

We conducted an epidemiologic study of thyroid cancer in women aged 40 and under to test the hypothesis that the development of thyroid cancer in these women is related to endogenous hormones – a hypothesis derived from a model we recently discussed at length (Henderson *et al.*, 1982). The only known cause of thyroid cancer, ionizing radiation (Hemplemann *et al.*, 1975; Favus *et al.*, 1976; Ron & Modan, 1980; Prentice *et al.*, 1982), does not account for the striking female excess of this disease. Data from cancer registries throughout the world show that thyroid cancer incidence is consistently 2 to 3 times higher in women than in men and that the incidence is similar in girls and boys under age 10 and changes to a female-to-male ratio of about 3 in puberty (ages 10–19), stays at a ratio of about 3 until the female menopause and then declines steadily to a ratio of 1.5 at age 65 (Waterhouse *et al.*, 1982).

We hypothesized that elevated levels of endogenous female sex hormones lead to elevated levels of thyroid stimulating hormone (TSH), elevated levels of TSH promote hyperplasia of the thyroid and this in turn increases the risk of thyroid cancer. Since TSH increases in pregnancy we would expect pregnancies to increase the risk of thyroid cancer. We would also expect to find associations with other menstrual and reproductive variables. The study we report was designed to test this suggested association for thyroid cancer.

### Materials and methods

The patients were Los Angeles County white women (excluding women born outside the USA, Canada or Europe), aged 15 to 40 years, with histologically confirmed thyroid cancer first diagnosed during the years 1980 and 1981. The patients were identified by the University of Southern California Cancer Surveillance Program, the population-based cancer registry for Los Angeles County. As the questionnaire sought information on reproductive history and contraceptive use, we restricted the study to living patients. Only 2 patients who would otherwise have been eligible were deceased.

The Cancer Surveillance Program identified 135 eligible cases. Their attending physicians granted permission to contact 127 (94%) of these patients. We were unable to locate 7 patients, and 10 refused to be interviewed. We therefore obtained completed questionnaires on 110 (92% of those contacted or 81% of all eligible cases).

We sought an individually matched neighbourhood control for each of the 110 cases. These controls had to be white women (with exclusions as above), and with birth dates within 5 years of their matched case. They also had to be at least as old at interview as their matched case was at diagnosis.

To find the neighbourhood controls we used a procedure that defines a sequence of houses on specified neighbourhood blocks and our goal was to interview the first matching female resident in the sequence (this procedure produces a close match between case and control on socioeconomic status) (Preston-Martin *et al.*, 1980). If no one was at home at the time of visit, we left an explanatory letter and made a follow-up visit after several days. In 98 instances, the first appropriate person agreed to cooperate. When the first matched control refused to participate the next in the sequence was sought. For any patient, 80 housing units were visited and 3 return visits made before failure to secure a matched control was conceded. In all, 108 matched neighbourhood controls were found and questionnaires completed.

All interviews were conducted by telephone by two interviewers; both members of a matched pair were interviewed by the same interviewer. Because we explained to each subject how we obtained her name, the interviewer was not blinded as to the subject's status. Interview information was obtained up to the present but analysis was limited for each case to events which occurred at least 1 year before diagnosis (reference date), and for each control to events which occurred before she reached the age that her matched case was at reference date (reference age). The case who had not begun to menstruate as of the reference date was excluded from the analysis of reproductive factors.

The matched pair design was maintained for the analysis of prior history of thyroid hyperplasia. However, because of the strength of this risk factor and the low prevalence in the controls (2/108), subjects with prior history of thyroid hyperplasia were eliminated from further analyses. To maximize sample size the data were then analyzed unmatched. In these unmatched analyses we tried various methods of adjusting for age. Since age adjustment in the analyses had little effect, we chose to report the unadjusted relative risk (RR) estimates. Unconditional logistic regression

Correspondence: S. Preston-Martin.
Received 25 June 1986; and in revised form, 7 October 1986.

methods were used to determine whether there was a dose-related increase or decrease in risk. In trend tests factors were always considered as continuous rather than as categorical variables. These multivariate methods were also used to examine the joint effects of several variables. All statistical methods used are described in detail in Breslow and Day (1980). All statistical significance levels (*P* values) quoted are 2-sided and exact 95% confidence intervals are shown.

### Results

Table I shows the distribution of cases by the histologic type of their tumours. All but 4 of the 108 cases had tumours classified as papillary, follicular or mixed papillary and follicular; more than half of the tumours were papillary (57 cases). The distribution of the cases' ages at diagnosis is also shown in Table I. Controls were matched to cases by year of birth (within 5 years), and the year of birth distribution of the controls was very similar to that of the cases.

Table II compares cases and controls on thyroid disease variables. Thyroid cancer was strongly related both to thyroid enlargement as an adolescent and to hyperplastic thyroid disease including goitre (11 cases; 2 controls) and benign nodules (11 cases; 2 controls). Subjects were counted as having adolescent thyroid enlargement or other thyroid disease only if a physician had been consulted and the diagnosis had been made at least 2 years before the case's cancer diagnosis. We combined these 2 variables to create an indicator of underlying hyperplastic thyroid disease (29 cases; 2 controls; RR = 14.5; $P<0.01$).

**Table I** Distribution by histologic type and age at diagnosis of women age 40 and under with thyroid cancer diagnosed in 1980–1981, Los Angeles County, California

| *Histologic type* | *Number of cases* |
|---|---|
| Papillary | 57 |
| Follicular | 15 |
| Mixed papillary/follicular | 32 |
| Other | 4 |
| All histologies | 108 |
| *Age at diagnosis* | |
| 15–19 | 15 |
| 20–24 | 16 |
| 25–29 | 34 |
| 30–34 | 26 |
| 35–40 | 17 |
| All ages | 108 |

Only 5 cases and 3 controls had a history of radiation treatment to the head or neck. All of these 5 cases, but only 1 control, had this treatment as a child or teenager (RR = 5.0; $P=0.14$). One of the other 2 controls had treatment for acne at age 24 and the other for Graves' disease (i.e. exophthalmic goitre) at age 23. Two cases had thymic irradiation as infants; 2 had adenoid irradiation, one at age 6 and the other at age 8; radiation treatment for acne was administered to 1 case at age 17 and to 1 control at age 17. All subjects estimated that they had 2 to 4 treatments except the 3 who had acne who estimated that they had 52 (control), 39 (case) and 13 (control) treatments. Cases and controls were not different in exposure to dental X-rays but 70 controls compared to only 58 cases stated that they had usually been protected by a lead apron up to the neck during dental radiography (RR = 0.6; $P=0.10$).

Table III compares cases and controls on various reproductive factors after excluding from the analysis the 29 cases and 2 controls with a history of hyperplastic thyroid disease. No trend is apparent relating age at menarche to risk of developing thyroid cancer. More cases than controls had never had regular menstrual cycles (RR = 1.6; $P=0.39$). More cases than controls had ever been pregnant (RR = 2.0; $P=0.04$), ever had a live birth (RR = 1.4; $P=0.29$), and ever had an incomplete pregnancy (RR = 1.9; $P=0.04$).

Thyroid cancer risk increased with increasing number of pregnancies (*P* in test for trend $<0.01$), and a significant increase in risk was associated with having 3 or more pregnancies (RR = 3.0; $P<0.01$). Age at first livebirth was not clearly related to risk.

Outcome of first pregnancy, in particular miscarriage (RR = 11.5; $P<0.01$), was strongly related to disease status. Elevated risk was also observed for ever having a miscarriage (RR = 2.7; $P=0.02$). Six of the 13 cases (and 0 of the 2 controls) whose first pregnancy ended in a miscarriage had subsequent miscarriages. Among women whose first pregnancy had some other outcome 6 cases and 8 controls had a later pregnancy which ended in miscarriage.

More cases than controls had ever used oral contraceptives (RR = 2.4; $P=0.02$). Cases and controls who ever used oral contraceptives (OCs) were not different with respect to age at first use. Women who used OCs for 24 months or less had increased risk compared to women who never used them (RR = 3.8; $P<0.01$). More cases than controls had ever stopped using OCs because they wanted to get pregnant (37 cases; 14 controls) and among short-term users (1–24 months) 17 cases and 2 controls stopped to try to get pregnant. Among the 17 cases and 2 controls who were short-term OC users and stopped using OCs because they wanted to get pregnant, 14 cases and 1 control used OCs before their first pregnancy; only 2 cases (out of these 14 cases and 1 control) never became pregnant.

Table IV shows that the total number of pregnancies has an independent effect on risk after thyroid disease and a miscarriage as the outcome of first pregnancy are taken into

**Table II** Comparison of young women with thyroid cancer to neighbourhood controls on history of thyroid hyperplasia, Los Angeles County, 1980–81

| | | *Number of cases* | *Number of controls* | *Discordant pairs* + − | *Discordant pairs* − + | *RR* | *2-sided P value* | *95% CI* |
|---|---|---|---|---|---|---|---|---|
| Thyroid enlargement | no | 98 | 107 | | | | | |
| as adolescent | yes | 10 | 1 | 10 | 1 | 10.0 | 0.03 | 1.3–78.0 |
| Goitre or benign | no | 87 | 106 | | | | | |
| nodules | yes | 21 | 2 | 21 | 2 | 10.5 | <0.01 | 2.5–44.8 |
| Thyroid disease | no | 79 | 106 | | | | | |
| (either of above) | yes | 29 | 2 | 29 | 2 | 14.5 | <0.01 | 3.5–60.8 |

**Table III** Comparison of young women with thyroid cancer and neighbourhood controls on various reproductive factors, Los Angeles County, 1980–81[a]

| | | *Number of cases* | *Number of controls* | *RR*[a] | *2-sided P* | *95% CI* | *Trend test* |
|---|---|---|---|---|---|---|---|
| Age at menarche | ≦11 | 16 | 22 | 1.0 | | | 0.53 |
| | 12 | 25 | 27 | 1.3 | 0.57 | 0.5–3.2 | |
| | 13 | 24 | 26 | 1.3 | 0.58 | 0.5–3.2 | |
| | ≧14 | 13 | 31 | 0.6 | 0.24 | 0.2–1.6 | |
| Menstruated regularly | ever | 71 | 100 | | | | |
| | never | 7 | 6 | 1.6 | 0.39 | 0.5–6.2 | |
| Ever pregnant | no | 22 | 46 | | | | |
| | yes | 56 | 60 | 2.0 | 0.04 | 1.0–3.9 | |
| Ever had a live birth | no | 53 | 55 | | | | |
| | yes | 54 | 52 | 1.4 | 0.29 | 0.7–2.6 | |
| Ever had an incomplete | no | 46 | 78 | | | | |
| pregnancy | yes | 32 | 28 | 1.9 | 0.04 | 1.0–3.8 | |
| Ever had a miscarriage | no | 61 | 96 | | | | |
| | yes | 17 | 10 | 2.7 | 0.02 | 1.1–7.0 | |
| Ever had an induced | no | 56 | 88 | | | | |
| abortion | yes | 22 | 18 | 1.9 | 0.07 | 0.9–4.2 | |
| Number of pregnancies | 0 | 22 | 46 | 1.0 | | | <0.01 |
| | 1 or 2 | 27 | 40 | 1.4 | 0.34 | 0.7–3.1 | |
| | ≧3 | 29 | 20 | 3.0 | <0.01 | 1.3–7.0 | |
| Number of live births | 0 | 35 | 56 | 1.0 | | | 0.07 |
| | 1 or 2 | 30 | 41 | 1.2 | 0.63 | 0.6–2.3 | |
| | ≧3 | 13 | 9 | 2.3 | 0.08 | 0.8–6.8 | |
| Age at first live birth | <20 | 14 | 18 | 1.0 | | | 0.24 |
| | 20–23 | 13 | 19 | 0.9 | 0.80 | 0.3–2.7 | |
| | ≧24 | 16 | 13 | 1.6 | 0.37 | 0.5–4.9 | |
| Outcome of first pregnancy | | | | | | | |
| never pregnant | | 22 | 46 | 1.0 | | | |
| live or stillbirth | | 32 | 45 | 1.5 | 0.25 | 0.7–3.1 | |
| induced abortion | | 13 | 13 | 2.1 | 0.12 | 0.8–5.8 | |
| miscarriage | | 11 | 2 | 11.5 | <0.01 | 2.3–112.0 | |
| Ever used oral contraceptives | no | 11 | 30 | | | | |
| | yes | 67 | 76 | 2.4 | 0.02 | 1.1–5.7 | |
| Duration of use of oral | never | 11 | 29 | 1.0 | | | 0.44 |
| contraceptives | ≦24 mos | 29 | 20 | 3.8 | <0.01 | 1.5–10.8 | |
| | 25–60 mos | 16 | 32 | 1.3 | 0.56 | 0.5–3.8 | |
| | >60 mos | 22 | 24 | 2.4 | 0.06 | 0.9–6.9 | |

[a]Excludes non-menstruating case and women with a history of thyroid hyperplasia.

account. Thyroid cancer risk increases as the total number of pregnancies increases. The RR for 4 or more pregnancies is 6.3. ($P=0.03$). The effect was similar when the analysis used number of full-term pregnancies rather than total number of pregnancies. As might be expected, risk of thyroid cancer also increases as a function of increasing total number of months pregnant after prior thyroid disease and miscarriage as the outcome of first pregnancy are considered.

For the major risk factors which emerged we also did analyses by histologic type of the tumour, but these analyses were restricted somewhat by the relatively small number of cases with follicular carcinoma (14 cases). In general, the same risk factors appeared to be important for papillary and mixed papillary and follicular tumours and for follicular carcinoma.

### Discussion

Our clearest finding is that thyroid cancer in women aged 40 and younger is related to early underlying thyroid disease. It is unlikely that this finding is due to poorer recall among controls since the rate of adolescent thyroid enlargement in our controls is similar to that of children living in the Western United States who were evaluated by palpation of their thyroid glands (Rallison *et al.*, 1974). In this study we did not attempt to assess the reproducibility of the telephone interview or to verify results by examination of medical records. We have done this, however, for similar telephone interview studies and have found no suggestion of recall bias (Preston-Martin *et al.*, 1985).

After adjusting for thyroid disease in the analysis, miscarriage is strongly related to risk; but miscarriage as the outcome of the first pregnancy may be another indicator of underlying thyroid disease. It has been shown that women whose thyroids fail to respond normally to pregnancy are more likely to miscarry (Man *et al.*, 1951). The risk associated with induced abortion as the outcome of first pregnancy (RR=2.1) is consistent with the risk for live or stillbirth as the outcome of first pregnancy (RR=1.5). More data are needed in order to evaluate the association of thyroid cancer with induced abortion.

Only 5 cases and 3 controls had a history of radiation treatment to the head or neck, and 7 of these 8 who did were born before 1946. Two recent case-control studies which included cases up to age 80 at diagnosis, however, found a history of radiation treatment to the head still to be a major risk factor (Ron *et al.*, 1987; McTiernan *et al.*, 1984*a*). Our finding suggests that the proportion of thyroid cancers attributable to this exposure is not only small in our

**Table IV** Comparison of young women with thyroid cancer to neighbourhood controls on outcome of first pregnancy and (if first pregnancy was not a miscarriage) on total number of pregnancies, Los Angeles County, 1980–81

| | *Number of cases* | *Number of controls* | *RR*[a] | *2-sided P* | *95% CI* | *Tread test* |
|---|---|---|---|---|---|---|
| Never pregnant | 22 | 46 | 1.0 | — | — | |
| Outcome of first pregnancy | | | | | | |
| Miscarriage | 11 | 2 | 11.5 | <0.01 | 2.3–112.0 | |
| Other outcome: | | | | | | |
| Total number of pregnancies | | | | | | |
| 1–2 | 25 | 40 | 1.3 | 0.46 | 0.6–2.8 | 0.05 |
| 3 | 14 | 16 | 1.8 | 0.18 | 0.7–4.8 | |
| ≥4 | 6 | 2 | 6.3 | 0.03 | 1.0–66.7 | |

[a]Excludes non-menstruating case and women with a history of thyroid hyperplasia.

young study population but may decrease in the future. The emphasis of epidemiologic studies must shift, therefore, to the study of other factors.

Both recent case-control studies found thyroid cancer to be strongly related to a history of benign thyroid disease, in particular to goitre and benign nodules (Ron *et al.*, 1987; McTiernan *et al.*, 1984*a*). One of these studies included both men and women and analyzed reproductive factors separately for the female cases and controls who were under age 35 (38 cases; 76 controls) and those 35 and over (71 cases; 133 controls) (Ron *et al.*, 1987). This study, in contrast to ours, found a significant trend of increasing risk related to increasing age at menarche in the younger group. A significant association was also observed only in the younger group with parity compared to nulliparity (RR = 2.3) and with having ever had a miscarriage (RR = 3.7). No relationship with age at first birth or number of livebirths was seen in either group.

The other study included women ages 18 to 80 (183 cases; 394 controls) (McTiernan *et al.*, 1984*a,b*). Risk was increased among parous compared to nulliparous women (RR = 1.8); this increase was seen for each of the 3 major histologic types (papillary, follicular, mixed). Risk was not related to age at first birth and did not increase with increasing number of pregnancies, but these analyses, unlike ours, apparently were not controlled for underlying thyroid disease. No association was seen for age at menarche. Ever use of OCs increased risk for all tumour types, but this finding was significant only for follicular carcinoma. As in our study there was no trend relating thyroid cancer to duration of use, and the strongest association was with short-term use (1–11 months). In our study this finding appears to some extent to be explained by the fact that controls had fewer pregnancies which was related to their greater duration of OC use and by the fact that more cases than controls in our study stopped taking OCs because they wanted to get pregnant; this difference was most striking among short-term users (17 cases; 2 controls). We wonder if more cases than controls might have started taking OCs in an attempt to regulate their menstrual periods, but unfortunately in this study we did not ask women why they started taking OCs. We are, however, asking the reason for starting OCs in a large study of thyroid cancer currently being conducted.

The proportion of first pregnancies which ended in miscarriage was 3.2% (2/62) for our controls and 18.3% (13/71) for our cases. Rates of spontaneous abortion have been found to be lower for first than for subsequent pregnancies and to increase with increasing age over 20 (Stevenson *et al.*, 1959). In Belfast, Ireland during 1957, the miscarriage rate of women aged 20–24 who were pregnant for the first time was 6.6% This rate (6.6%) is higher than that in our controls (3.2%), but is also considerably lower than that in our cases (18.3%). It seems reasonable that the miscarriage rate for first pregnancies among young white women in Los Angeles County in the 1980s may be different from the rate among young women in Belfast in 1957. Although several surveys of foetal loss have been done no other has reported the miscarriage rate for women in their early twenties who were pregnant for the first time. The median age of our controls at their first pregnancy was 21.2 years. When all pregnancies are considered, 8.9% (12/135) of those among our controls ended in miscarriage compared to 11.8% in the Belfast cohort; this difference is largely attributable to the older age at pregnancy among the women in Belfast. In our study differential recall appears not to be a problem since, among women who ever had a livebirth, 22 of 54 cases and 21 of 52 controls reported having had at least one incomplete pregnancy.

The initial aim of our case-control study of thyroid cancer in young women was to test the hypothesis that these cancers are related to endogenous hormones. This hypothesis was derived from our model which proposes that individual hormones which usually control normal growth of target organs can cause neoplastic growth in that organ when hormone levels are excessive (Henderson *et al.*, 1982). TSH is the principal hormone regulating the growth and function of the thyroid gland (Ingbar & Woeber, 1974), and we therefore suggested a TSH excess hypothesis for thyroid cancer. This hypothesis is supported by the observation that growth of some thyroid cancers is dependent on TSH secretion so that suppression of TSH release by administration of thyroxin is often an effective treatment for thyroid carcinomas (Crile, 1966).

The hypothesis is also supported by experimental work. Sustained elevation of TSH induces thyroid tumours in rodents (Axelrad & Leblond, 1955; Griesback *et al.*, 1941) and the mechanism by which elevated TSH levels are achieved appears unimportant. Thyroid tumours have been produced by iodine deficient diets, by blocking thyroid hormone synthesis, by administering TSH directly, and by chemical goitrogens (Morris, 1954).

Increased levels of female sex hormones are associated with increased levels of thyroid hormones as seen in early pregnancy when a 50% increase in thyroxin-binding globulin (TBG) and in TSH are observed (Malkasian & Mayberry, 1970). TBG levels in non-pregnant females are 10 to 20% higher than in males (Gershengorn *et al.*, 1980). Changes in the size and activity of the thyroid during the course of a normal menstrual cycle have been observed (Robbins, 1979); these changes are likely to relate to transient elevations in TSH levels.

Our findings suggest that pregnancy increases thyroid cancer risk. After eliminating women with underlying thyroid disease and those whose first pregnancy ended in a miscarriage, we observed an increase in risk with an increasing total number of pregnancies. We present, therefore, support for the model that endogenous hormones increase the risk of thyroid cancer. The number of cases and controls studied was small, however, and the confidence limits around most risk estimates we derived were, therefore, quite wide.

This investigation was supported by PHS grant number CA 17054 awarded by the National Cancer Institute, DHHS.

**References**

AXELRAD, A.A., LEBLOND, C.P. (1955). Induction of thyroid tumors in rats by a low iodine diet. *Cancer*, **8**, 339.

BRESLOW, N.E. & DAY, N.E. (1980). *Statistical Methods in Cancer Research: The Analysis of case-control studies*, Volume I. IARC Scientific Publication; **32**, Lyon, France.

CRILE, G. (1966). Endocrine dependency of papillary carcinomas of the thyroid. *J.A.M.A.*, **195**, 721.

FAVUS, M.J., SCHNEIDER, A.B., STACHURA, M.E. & 6 others (1976). Thyroid cancer occurring as a later consequence of head-and-neck irradiation: Evaluation of 1056 patients. *N. Engl. J. Med.*, **294**, 1019.

GERSHENGORN, M.C., GLINOER, D. & ROBBINS, J. (1980). Transport and metabolism of thyroid hormones. In *The Thyroid Gland*, DeVisscher, M. (ed), Raven Press: New York.

GRIESBACK, W.E., KENNEDY, T.H. & PURVES, H.D. (1941). Studies on experimental goitre III. The effect of goitrogenic diet on hypophysectomized rats. *Br. J. Exp. Pathol.*, **22**, 249.

HEMPLEMANN, L.H., HALL, W.J., PHILLIPS, M., COOPER, R.A. & AMES, W.R. (1975). Neoplasms in persons treated with X-rays in infancy: Fourth survey in 20 years. *J. Natl Cancer Inst.*, **55**, 519.

HENDERSON, B.E., ROSS, R.K., PIKE, M.C. & CASAGRANDE, J.T. (1982). Endogenous hormones as a major factor in human cancer. *Cancer Res.*, **42**, 3232.

INGBAR, S.H. & WOEBER, K.A. (1974). The thyroid gland. In *Textbook of Endocrinology*, Williams, R.H. (ed), W.B. Saunders: Philadelphia.

MALKASIAN, G.D. & MAYBERRY, W.E. (1970). Serum total and free thyroxine and thyrotropin in normal and pregnant women, neonates, and women receiving progestogens. *Amer. J. Obstet. Gynec.*, **108**, 1234.

MAN, E.B., HEINEMANN, M., JOHNSON, C.E., LEARY, D.C. & PETERS, J.P. (1951). The precipitable iodine of serum in normal pregnancy and its relation to abortions. *J. Clin. Invest.*, **30**, 137.

McTIERNAN, A.M., WEISS, N.S. & DALING, J.R. (1984*a*). Incidence of thyroid cancer in women in relation to previous exposure to radiation therapy and history of thyroid disease. *J. Natl Cancer Inst.*, **73**, 575.

McTIERNAN, A.M., WEISS, N.S. & DALING, J.R. (1984*b*). Incidence of thyroid cancer in women in relation to reproductive and hormonal factors. *Am. J. Epidemiol.*, **120**, 423.

MORRIS, H.P. (1954). Experimental thyroid tumors. *Brookhaven Symposia in Biology*, **7**, 192.

PRENTICE, R.L., KATO, H., YOSHIMOTO, K. & MASON, M. (1982). Radiation exposure and thyroid cancer incidence among Hiroshima and Nagasaki residents. In *Third Symposium on Cancer Registries in the Pacific Basin*. NCI Monograph, **62**, 207.

PRESTON-MARTIN, S., BERNSTEIN, L., MALDONADO, A.A., HENDERSON, B.E. & WHITE, S.C. (1985). A dental X-ray validation study: Comparison of information from patient interviews and dental charts. *Am. J. Epidemiol.*, **121**, 430.

PRESTON-MARTIN, S., PAGANINI-HILL, A., HENDERSON, B.E., PIKE, M.C. & WOOD, C. (1980). Case-control study of intracranial meningiomas in women in Los Angeles County. *J. Natl Cancer Inst.*, **65**, 67.

RALLISON, M.L., DOBYNS, B.M., KEATING, F.R., RALL, J.E. & TYLER, F.H. (1974). Thyroid disease in children. *Am. J. Med.*, **56**, 457.

ROBBINS, S.L. (1979). The thyroid gland. In *Pathologic Basis of Disease*, Robbins, S.L. & Cotran, R.S. (eds), W.B. Saunders: Philadelphia.

RON, E., KLEINERMAN, R.A., BOICE, J.D., LIVOLSI, V.A. & FLANNERY, J.T. (1987). Risk factors for thyroid cancer in Connecticut: A population-based case-control study.

RON, E. & MODAN, B. (1980). Benign and malignant thyroid neoplasms after childhood irradiation for tinea capitis. *J. Natl Cancer Inst.*, **65**, 7.

STEVENSON, A.C., DUDGEON, M.Y. & McCLURE, H.I. (1959). Observations on the results of pregnancies in women in Belfast. II. Abortions, hydatidiform moles and ectopic pregnancies. *Ann. Hum. Genet.*, **23**, 395.

WATERHOUSE, J., MUIR, C., SHANMUGARATNAM, K. & POWELL, J. (eds) (1982). *Cancer Incidence in Five Continents*, Vol. IV. IARC Scientific Publications; **42**, Lyon, France.